# CD19 and CD30 CAR T-Cell Immunotherapy for High-Risk Classical Hodgkin’s Lymphoma

**DOI:** 10.3389/fonc.2020.607362

**Published:** 2021-02-02

**Authors:** YuanBo Xue, Xun Lai, RuiLei Li, ChunLei Ge, BaoZhen Zeng, Zhen Li, QiaoFen Fu, LiuFang Zhao, SuWei Dong, JinYan Yang, JiYin Guo, QingYin Meng, QingHua Tan, ZhenHui Li, HaiYan Ding, YanLei Zhang, ShaoHui Liu, Alex H. Chang, Hong Yao, RongCheng Luo

**Affiliations:** ^1^Cancer Center, Integrated Hospital of Traditional Chinese Medicine, Southern Medical University Guangdong, Guangzhou, China; ^2^Cancer Biotherapy Center, The Third Affiliated Hospital of Kunming Medical University (Tumor Hospital of Yunnan Province), Kunming, China; ^3^Department of Hematology, The Third Affiliated Hospital of Kunming Medical University (Tumor Hospital of Yunnan Province), Kunming, China; ^4^Department of Head and Neck Surgery, The Third Affiliated Hospital of Kunming Medical University (Tumor Hospital of Yunnan Province), Kunming, China; ^5^Department of Osteology, The Third Affiliated Hospital of Kunming Medical University (Tumor Hospital of Yunnan Province), Kunming, China; ^6^Department of Pathology, The Third Affiliated Hospital of Kunming Medical University (Tumor Hospital of Yunnan Province), Kunming, China; ^7^Department of Radiology, The Third Affiliated Hospital of Kunming Medical University (Tumor Hospital of Yunnan Province), Kunming, China; ^8^Clinical Translational Research Center, Shanghai Pulmonary Hospital, Tongji University School of Medicine, Shanghai, China

**Keywords:** immunotherapy, classical Hodgkin’s lymphoma (cHL), CD19, CD30, chimeric antigen receptor (CAR) T-cell

## Abstract

**Background:**

In clinical applications of CAR T-cell therapy, life-threatening adverse events including cytokine release syndrome and neurotoxicity can lead to treatment failure. Outcomes of patients treated with anti-CD30 CAR T- cell have been disappointing in relapsing/refractory (r/r) classical Hodgkin’s Lymphoma (cHL).

**Methods:**

In order to understand the applicable population of multiple CAR T-cell therapy, we examined the expression of CD19, CD20, and CD30 by immunohistochemistry (IHC) in 38 paraffin-embedded specimens of cHL. In the past two years, we found only one patient with cHL who is eligible for combined anti-CD19 and CD30 CAR T-cell treatment. This patient’s baseline characteristics were prone to severe adverse events. We treated this patient with low doses and multiple infusions of anti-CD19 and CD30 CAR T-cell.

**Results:**

The positive expression of CD19^+^ + CD30^+^ in Reed-Sternberg (RS) cells is approximately 5.2% (2/38). The patient we treated with combined anti-CD19 and CD30 CAR T-cell did not experience severe adverse events related to CAR T-cell therapy and received long term progression-free survival (PFS).

**Conclusion:**

For high risk r/r cHL patients, low doses of CAR T-cell used over different days at different times might be safe and effective. More clinical trials are warranted for CD19 and CD30 CAR T-cell combination therapy.

## Introduction

Classical Hodgkin’s Lymphoma (cHL) can be cured using standard chemotherapy with or without radiation (1). Despite multi-modal therapy, 15–20% of patients with cHL succumb to this disease (2). The prognosis for patients with cHL that are relapsing/refractory (r/r) after first-line treatment or autologous stem cell transplantation (ASCT) is very poor (3). About 10–40% of patients do not achieve a response to salvage chemotherapy and no randomized clinical trial data support ASCT in non-responders (4). Therefore, it is imperative to develop novel approaches to improve the prognosis for patients with r/r cHL.

Hodgkin’s lymphoma cells (mononucleated giant cell) or Reed-Sternberg (RS) cells are a typical morphological feature of cHL (5). The expression of CD30 molecules in RS cells is greater than 98% (1). Besides CD30, some B cells antigens, such as CD19 and CD20, have been identified in cHL (6). Although CD30 is very lowly expressed in normal tissues, it is selectively overexpressed in RS cells, rendering this antigen a promising target for novel treatment strategy (3).

Chimeric antigen receptor (CAR) T-cell therapy is an effective method for treating certain cancers (7). CARs are normally designed to recognize those antigens that are highly expressed in malignant cells (7–9). CAR combines an extracellular antigen-binding domain of an antibody (scFv) with a transmembrane domain, linked to one or more intracellular T-cell signaling domains (3). Unfortunately, clinical trial outcomes in r/r cHL patients treated with anti-CD30 CAR T-cell have been disappointing (2, 3). As if yet, there is no clinical trial with CAR T-cell combination therapy for cHL. Currently, CD19 CAR T-cell have been approved by the FDA for the treatment of acute lymphoblastic leukemia (ALL) and diffuse large B cell lymphoma (DLBCL) (10). We hypothesized that if CD19 and CD30 are positively expressed in patient tumor RS cells, a combination of anti-CD19 and CD30 CAR T-cells may be an effective therapy for the treatment of r/r cHL. To understand which CAR T-cell could be used for the treatment of cHL and how many cHL patients could receive multiple CAR T-cell therapy, we detected the expression of CD19, CD20, and CD30 in RS cells by IHC staining in 38 paraffin-embedded specimens from patients with cHL.

A number of CAR T-cell clinical trials have shown that life-threatening adverse events such as cytokine release syndrome (CRS) and neurotoxicity (NT) (11) might lead to treatment failure (7). Current treatment strategies have been focused on improving the safety of CAR-T therapy (7).

In this study, we recruited a patient with refractory cHL who had a high tumor burden, severe thrombocytopenia, and poor physical condition. We treated the patient multiple times with a low does of CD19 and CD30 CAR T-cell combination therapy. Excitingly, this patient did not experience severe adverse events related to CAR T-cell therapy. More importantly, the patient received a long time progression-free survival (PFS).

## Methods and Patients

### Study Design

We recruited patients with cHL for this phase I/II single-center clinical trial that met the following inclusion criteria: 1) 18 to 75 years old with CD19^+^ and CD30^+^ r/r lymphoma confirmed by IHC staining; 2) Eastern Cooperative Oncology Group (ECOG) performance status of 2 or less; 3) measurable lesion ≥1 cm; 4) previous treatment with at least 2 systemic chemotherapy regimens concluded at least 4 weeks prior; 5) no ASCT within 12 weeks; and 6) passing the expert panel discussion. The exclusion criteria were 1) severe organ dysfunction; 2) a history of active systemic autoimmune/immunodeficiency disease; and 3) treatment history of immunosuppressive agents or glucocorticoids within the last month. All patients provided written informed consent before enrolling in the study. The study was approved by the ethics committee of The Third Affiliated Hospital of Kunming Medical University, and followed the Declaration of Helsinki. Trial registration: http://www.chictr.org.cn, Trial number: ChiCTR2000028922.

### Lentiviral Construction and Preclinical Evaluation of Chimeric Antigen Receptor T-Cell

As previously described, lentiviral vectors (LV) were generated based on the NHP/TYF system (12). The CAR containing CD19 or CD30-specific scFv fused with a 4-1BB co-stimulatory, and CD3ζsignaling domains (scFv/4-1BB/CD3ζ), was chemically synthesized and cloned into pTYF transducing vector under the regulation of a human EF1α promoter (13). The scFv of the CD19 CAR is derived from a murine antibody, whereas the CD30 CAR has been humanized. The final LV-CAR construct was extensively verified by functional analyses. For the preparation of clinical grade CAR T-cell, a standard operating protocol has been established in compliance with good manufacturing and laboratory practices following regulatory guidelines for cell and gene therapy products (14).

### Generation of Chimeric Antigen Receptor T-Cell

The manufacturing of CAR T-cell from peripheral blood mononuclear cells (PBMCs) commenced on the day of leukapheresis and was completed in 10 days. PBMCs collected from the patient were stimulated with magnetic beads coated with anti-CD3/CD28 antibodies (Life Technologies, Carlsbad, CA, USA; now owned by Thermo Fisher Scientific, Waltham, MA, USA) overnight (13). The next day, transduction with recombinant lentiviral vectors of CD19 or CD30 was performed at a 1:10 multiplicity of infection. Transduced cells were cultured in X-VIVO 15, a serum-free medium (Lonza) with 300 IU/ml interleukin-2 (IL-2) (13). Transduction efficiency was defined as the ratio of CAR T-cell to CD3^+^ T cells determined by Flow Cytometry (FCM) with a proprietary anti-CD19 CD30 CAR-T cell-specific detection reagent (13). Cell viability was determined by Trypan blue exclusion (10). Transduction efficiency and cell viability were examined before cell infusion (13).

### Lymphodepletion Chemotherapy and Chimeric Antigen Receptor T-Cell Infusion

To deplete endogenous lymphocytes before adoptive transfer of CAR T-cell, the patient received chemotherapy regimens of both cyclophosphamide (Cy) 0.5g IV and fludarabine (Flu) 50 mg IV on day 1 to day 3. After 96 h of Cy/Flu-based partial myeloablation chemotherapy, CAR T-cell were infused intravenously.

### Flow Cytometry

CD19, CD20, and CD30-positive cells were analyzed from the patient’s lymphoma biopsy material using three-color Flow Cytometry (FCM) before CAR T-cell infusion. Briefly, the biopsy tissue was cut into small pieces and digested by pancreatin to make a single-cell suspension. Cell suspensions were then incubated with mixtures of antibodies specific to CD19, CD20, and CD30 (15). The antibodies were directly conjugated with allophycocyanin (APC), phycoerythrin (PE), or peridinin chlorophll protein (PERCP). FCM analysis was performed using a COULTERs EPICS XLt flow cytometer (Beckman Coulter, Miami, FL, USA) (15). CAR T-cell were determined by FCM with a proprietary anti-CD19, CD30, and CAR-T cell-specific detection reagent.

We used the BD CBA Human Soluble Protein Flex Set System to determine plasma concentrations of IL-2, IL-4, IL-6, and IL-10 cytokines, tumor necrosis factor α(TNF-α), and interferon-γ (IFN-γ). Using FCM, the CBA system captures soluble analytes with beads of known size and fluorescence (14).

### Immunohistochemical Staining

To understand the applicable population and possibility of combination CAR T-cell therapy, we detected the expression of CD19, CD20, and CD30 in RS cells by immunohistochemical (IHC) staining in 38 paraffin specimens from patients with cHL. These cHL specimens were collected by the department of pathology from 2018 to 2019. The median age of patients was 40 (range 17–82), including 22 males and 16 females. Each paraffin specimen was immunohistochemically stained with antibodies against CD19, CD20, and CD30 using mouse monoclonal antibodies. These 38 specimens included 2 Lymphocytic Hodgkin’s lymphoma, 16 mixed cellularity Hodgkin’s lymphoma, 14 nodular sclerosis Hodgkin’s lymphoma, and 2 lymphocyte-rich Hodgkin’s lymphoma. Four specimens could not be classified. All IHC staining results were defined as negative or positive.

The recruited patient’s paraffin sections from the metastatic lymphoma biopsy material were prepared for H&E staining and CD19 and CD30 IHC staining before CAR T-cell therapy.

## Results

### The Expression of CD19, CD20, and CD30 in Patients With Classical Hodgkin’s Lymphoma

To evaluate the expression of CD19, CD20, and CD30 in patients with cHL, immunohistochemical staining using antibodies against CD19, CD20, and CD30 was performed. Among the 38 paraffin-embedded cHL specimens, only 2 (5.2%) specimens showed positive CD19 staining in RS cells, including 1 mixed cellularity Hodgkin’s lymphoma and 1 unknown case. There were 37 (97.3%) specimens that showed positive CD30 staining and 31 (81.5%) specimens that showed positive CD20 staining in RS cells. There were 28 (73.6%) specimens that showed both CD30 and CD20 positive staining in RS cells. Two (5.2%) specimens had positive CD19 + CD30 and CD19 + CD20 staining in RS cells. Two (5.2%) specimens had positive CD19 + CD20 + CD30 staining in RS cells (**Table 1**).

**Table 1:** CD19, CD20, and CD30 Immunohistochemical (IHC) staining in classical Hodgkin’s lymphoma (cHL) paraffin-embedded specimens.

Case No	Gender	Age	Pathological type	CD20	CD30	CD19
01	Male	57	lymphocyte-rich	+	+	–
02	Female	39	lymphocyte-rich	+	+	–
03	Male	55	Lymphocytic	+	+	–
04	Male	17	Lymphocytic	+	+	–
05	Male	43	difficult to classify	+	+	+
06	Male	33	difficult to classify	+	+	–
07	Male	53	difficult to classify	+	+	–
08	Female	76	difficult to classify	+	+	–
09	Male	82	mixed cellularity	+	+	+
10	Male	18	mixed cellularity	+	–	–
11	Male	23	mixed cellularity	+	+	–
12	Male	53	mixed cellularity	+	+	–
13	Male	18	mixed cellularity	+	+	–
14	Male	42	mixed cellularity	+	+	–
15	Male	22	mixed cellularity	–	+	–
16	Male	32	mixed cellularity	–	+	–
17	Male	62	mixed cellularity	–	+	–
18	Male	75	mixed cellularity	+	+	–
19	Female	37	mixed cellularity	+	–	–
20	Female	55	mixed cellularity	+	+	–
21	Female	69	mixed cellularity	+	+	–
22	Female	41	mixed cellularity	+	+	–
23	Female	63	mixed cellularity	+	+	–
24	Female	20	mixed cellularity	+	+	–
25	Male	39	nodular sclerosis	+	+	–
26	Male	21	nodular sclerosis	+	+	–
27	Male	68	nodular sclerosis	–	+	–
28	Male	32	nodular sclerosis	+	+	–
29	Male	59	nodular sclerosis	–	+	–
30	Male	32	nodular sclerosis	–	+	–
31	Female	38	nodular sclerosis	+	+	–
32	Female	29	nodular sclerosis	+	+	–
33	Female	25	nodular sclerosis	–	+	–
34	Female	38	nodular sclerosis	+	+	–
35	Female	23	nodular sclerosis	+	+	–
36	Female	27	nodular sclerosis	+	+	–
37	Female	19	nodular sclerosis	+	+	–
38	Female	34	nodular sclerosis	+	+	–

“+” Indicates positive expression in Reed-Sternberg (RS) cells ; “-” Indicates no expression in Reed-Sternberg (RS) cells.

### The Expression of CD19, CD20, and CD30 in the Recruited Patient With Classical Hodgkin’s Lymphoma

**Figure 1:**
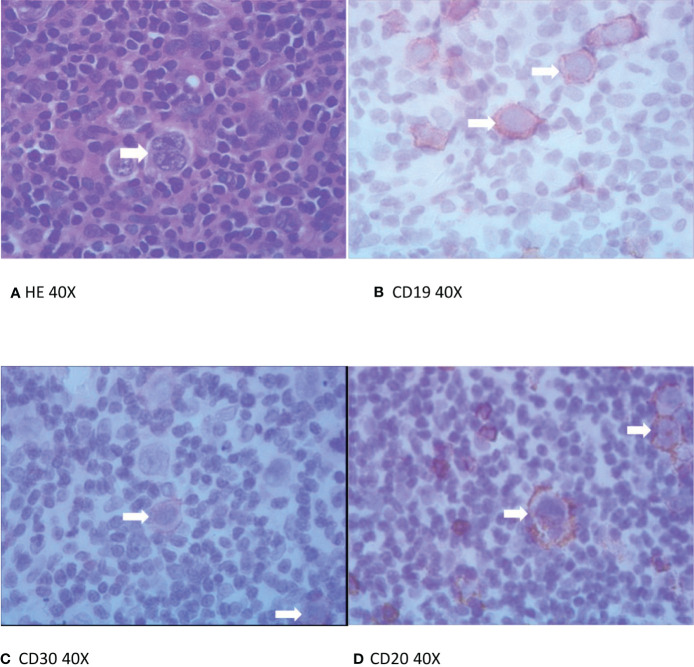
Hematoxylin and Eosin staining (H&E) and immunohistochemical (IHC) staining of paraffin specimens. **(A)** H&E, staining in Reed-Sternberg cells (arrows). **(B–D)** of CD19, CD30, and CD20 IHC staining, respectively, in Reed-Sternberg cell (arrows).

The expression of CD19, CD20, and CD30 in the metastatic lymphoma tissue of the recruited patient was evaluated. IHC staining showed positive CD19 and CD30 staining in RS cells (**Figure 1**).

**Figure 2:**
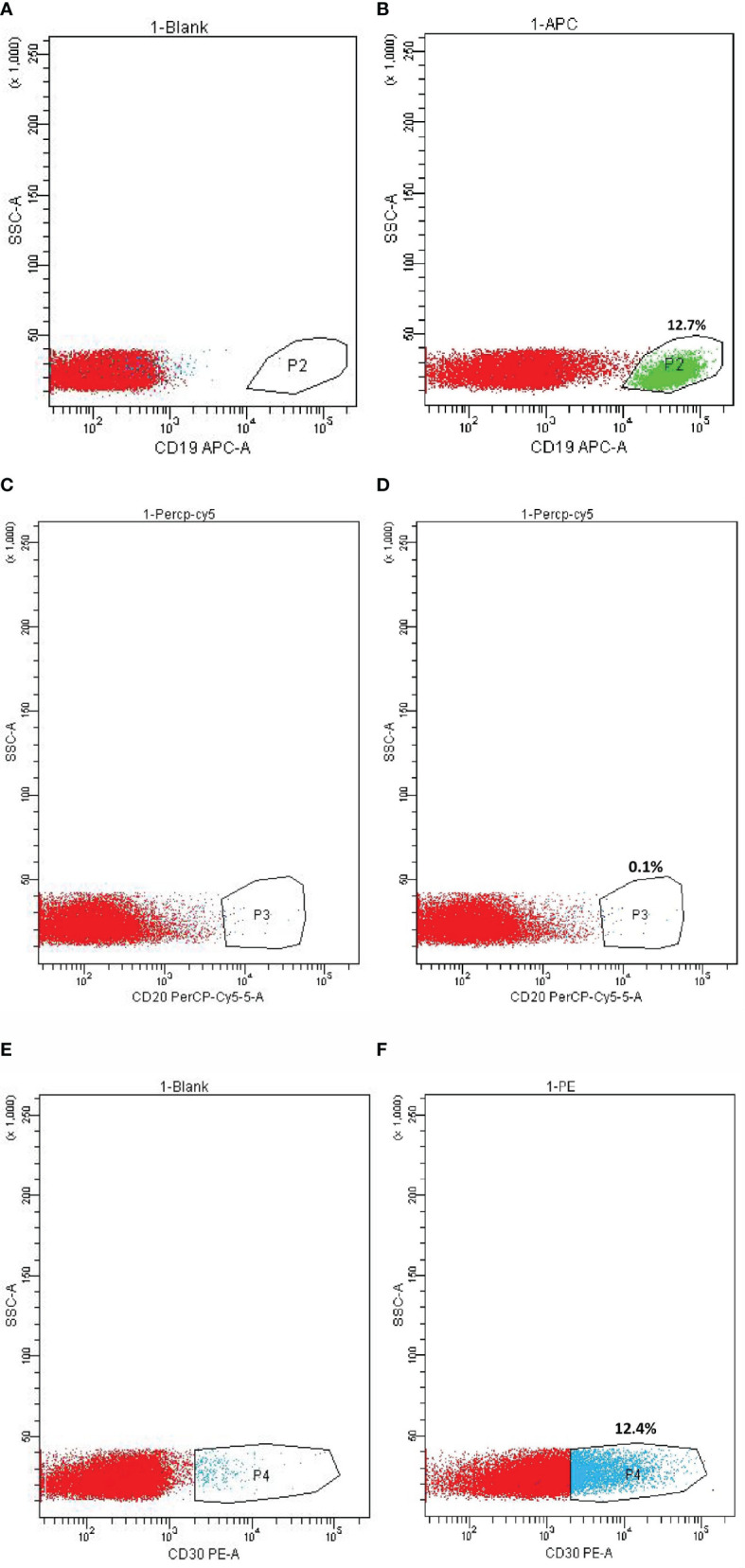
Flow Cytometry (FCM) on CD19, CD20, and CD30 expression in metastatic lymphoma tissue. **(A)** CD19 expression cell control. **(B)** CD19-positive cells. **(C)** CD20 expression cell control. **(D)** CD20-positive cells. **(E)** CD30 expression cell control. **(F)** CD30-positive cells.

The percentage of CD19, CD20, and CD30-positive cells in the lymphoma tissue was analyzed by FCM (**Figure 2**), which showed 12.7, 0.1, and 12.4% of positive cells, respectively.

### Patient’s Characteristics and Chimeric Antigen Receptor T-Cell Therapy

**Figure 3:**
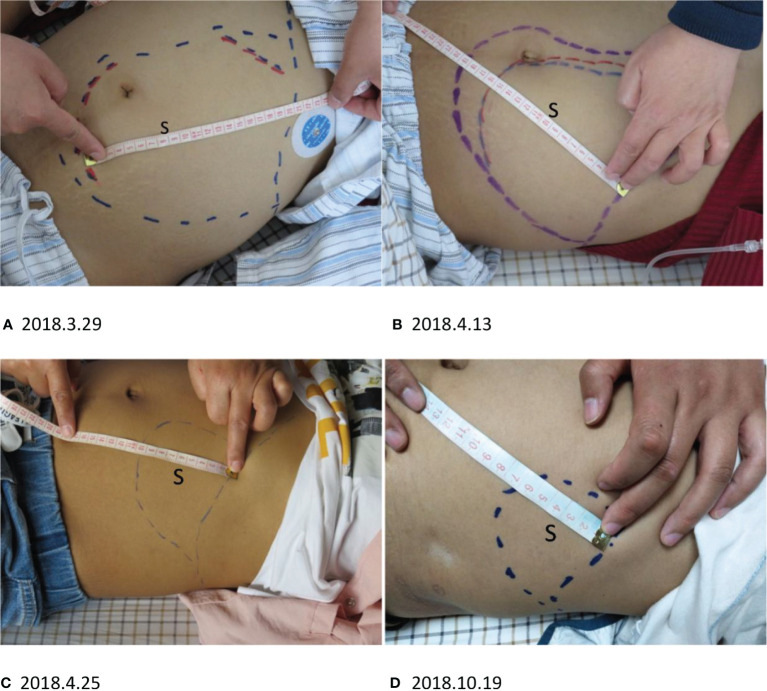
S, splenomegaly. **(A–C)** The splenomegaly ameliorated quickly after CD19 CAR T-cell infusion. **(A)** The outermost blue line is the margin of splenomegaly before CD19 CAR T-cell treatment. The red line indicates the splenomegaly length after three days. **(B)** The splenomegaly shrank in approximately one month. The innermost blue line indicates the margin of the spleen two weeks after CAR T-cell infusion. **(C)** The splenomegaly continued to shrink before CD30 CAR T-cell infusion. **(D)** After CD30 CAR T-cell infusion, the splenomegaly continued to improve.

The patient is a 21-year-old female who had been primarily diagnosed with cHL (nodular sclerosis) IV_5E_ B_lshoM-_, international prognostic index (IPI) 3, ECOG performance status 2. She had previously received five cycles of first-line systemic chemotherapy using ABVD (doxorubicin, bleomycin, vinblastine, and dacarbazine). Following, computed tomography (CT) analysis showed stable disease (SD) as compared to the previous analysis the patient received after two cycles of systemic chemotherapy. For economic reasons, she declined second-line systemic chemotherapy, and received no treatment for ten months. Approximately two months before CAR T-cell therapy, the patient started experiencing hyperpyrexia (38.5-41.0°C). Approximately three weeks before CAR T-cell therapy, CT scans showed cancer cells had metastasized to the spleen, liver, lung, bone, and lymph nodes located in the neck, chest, abdomen, and pelvis. One week before CAR T-cell administration, the patient displayed obvious recurring symptoms, including hypotension (97-86/54-63 mmHg), increased heart rate (120-144 times/min), dry cough, anorexia, nausea, vomiting, malaise, fatigue, headaches, hallucinations, and splenomegaly over the midline (**Figure 3A**). According to the Common Terminology Criteria for Adverse Events (CTCAE) 4.0 rating criteria: cough and hallucinations are grade 1; anorexia, nausea, vomiting, and malaise are grade 2; fatigue and headaches are grade 3. These symptoms are tumor complications because there was no abnormality found in cerebrospinal fluid examination. Labwork showed hemoglobin of 5.9 g/dL, platelet of 55×10^9^/L, coagulopathy, serum C-reactive protein (CRP) of 114.26 mg/L, and interleukin-6 (IL-6) of 146.69 pg/ml. At this point, the patient would likely only have a few weeks to live if she had declined to be treated. Because CD19 and CD30 were overexpressed in RS cells of the patient’s metastastatic lymphoma, we recruited this patient to participate in the CAR T-cell clinical trial. With the fully informed consent of the patient and her family, we combined anti-CD19 and CD30 CAR T-cell treatment for this patient.

After 96 h Cy/Flu-based partial myeloablation chemotherapy, CAR T-cell were infused intravenously. Since the patient’s baseline characteristics were prone to severe adverse events, we attempted to reduce the risk of acute toxicities such as CRS and NT. We split the total dosage of CD19 CAR T-cell into three parts and administered the cells once every six days (16–18). The first infusion was performed at a low dose (1.61×10^5^ cells/kg) and there was no serious adverse event. The second and third doses of anti-CD19 CAR T-cell were conducted using a dose of 5.20×10^5^cells/kg. There was no serious adverse events from CD19 CAR T-cell therapy. Twenty-four days after the last CD19 CAR T-cell infusion there were no CD19 CAR T-cell detected.

**Figure 4:**
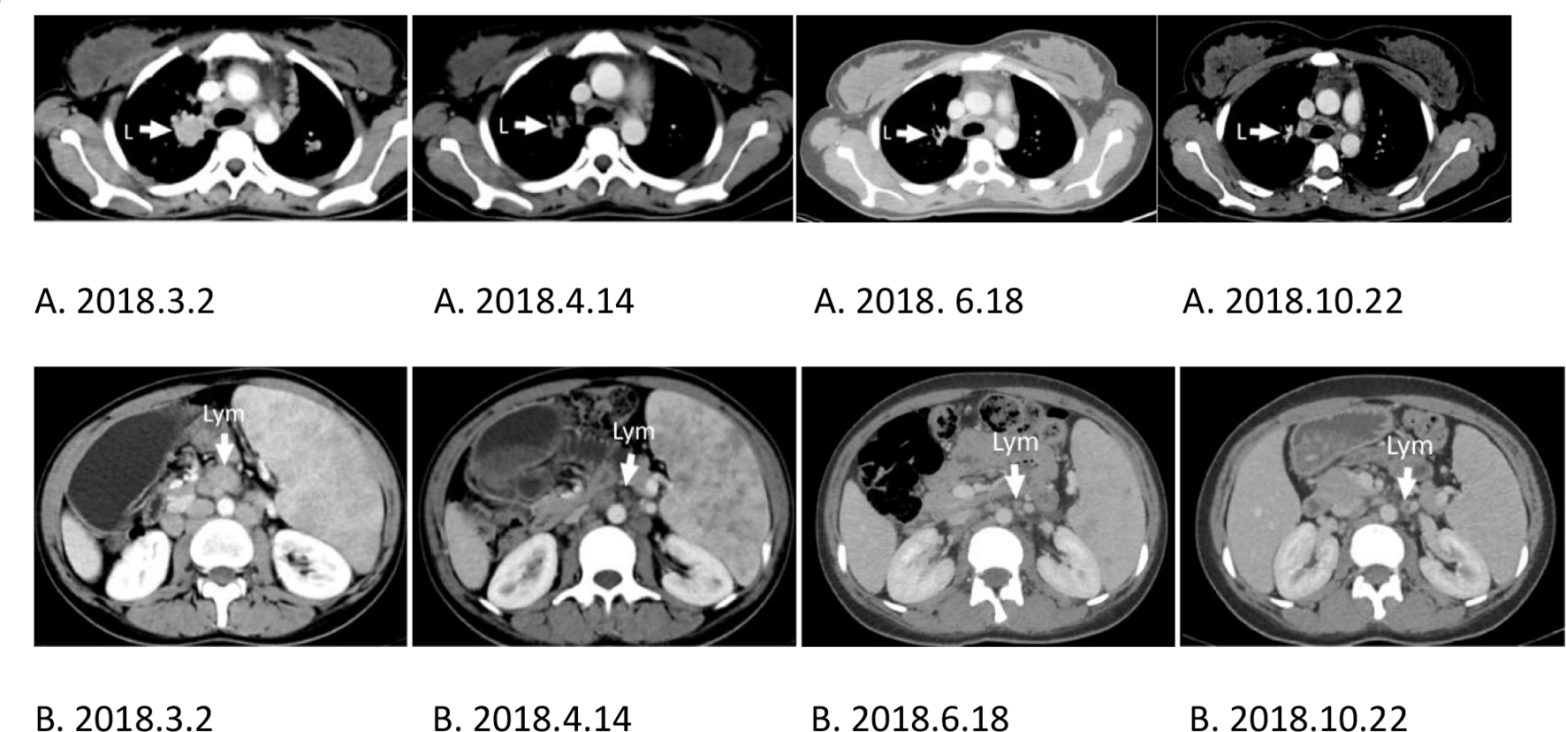
L, Lung; Lym, lymphonodus. **(A, B)** CT scans comparing the same location as indicated by the arrow at different times showing tumor reduction. **(A)** Lung metastasis. **(B)** Lymphadenopathy of the abdomen. **(A, B)** 2018.3.2. The baseline of lung metastasis or lymphadenopathy of the abdomen before CD19 CAR T-cell treatment. **(A, B)** 2018.6.18. The baseline of lung metastasis or lymphadenopathy of the abdomen before CD30 CAR T-cell treatment.

After three days of the first CD19 CAR T-cell therapy, the patient’s physical condition improved. The conditions of hyperpyrexia and hypotension subsided, her heart rate returned to normal, and her dry cough, anorexia, nausea, vomiting, malaise, fatigue, headaches, and hallucinations were significantly reduced, completely disappearing within one week. Two weeks after the first CD19 CAR T-cell infusion, the splenomegaly shrank quickly (**Figures 3A, B**), and continued to shrink before CD30 CAR T-cell infusion (**Figure 3C**). Approximately two weeks after the first CD19 CAR T-cell infusion, CT scans showed significant shrinkage of spleen and lung metastases (**Figure 4A** 2018.4.14), and lymphadenopathy in the neck, abdomen (**Figure 4B** 2018.4.14), and pelvis.

**Figure 5:**
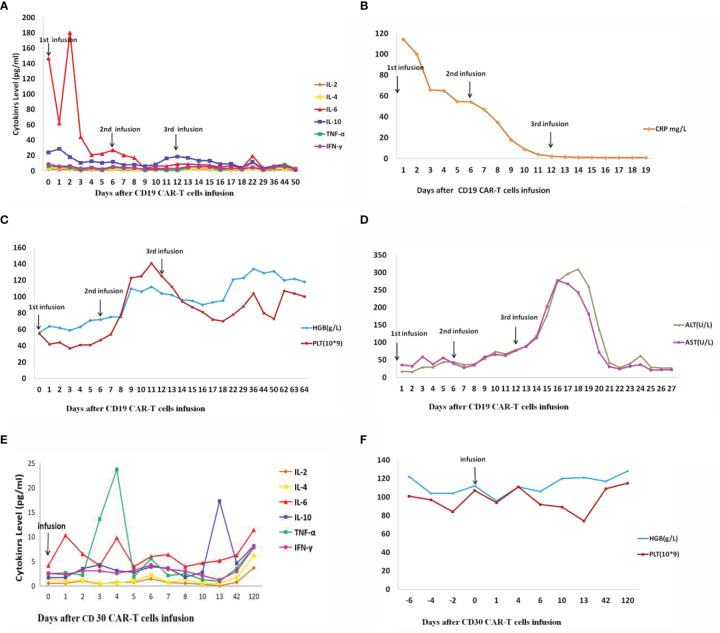
IL, interleukin; TNF, tumor necrosis factor; IFN, interferon; CRP, C-reactive protein; HGB, hemoglobin; PLT, platelet; ALT, alanine transaminase; AST, Aspartate transaminase. **(A–D)** Changes in cytokines and other factors after three times(1st, 2nd, and 3rd) CD19 CAR T-cell infusion. **(E, F)** Changes in cytokines and other factors after CD30 CAR T-cell infusion. **(A)** Cytokines change after CD19 CAR T-cell infusion. **(B)** CRP changes after CD19 CAR T-cell infusion. **(C)** HGB and PLT change after CD19 CAR T-cell infusion. **(D)** ALT and AST change after CD19 CAR T-cell infusion. **(E)** Cytokines change after CD30 CAR T-cell infusion. **(F)** HGB and PLT change after CD30 CAR T-cell infusion.

Lab results showed that the patient’s IL-6 (**Figure 5A**) transiently increased from 146.69 pg/ml to 180.37 pg/ml, and then gradually decreased to 2.83 pg/ml after two days of the first CD19 CAR T-cell infusion. The level of hemoglobin and platelets (**Figure 5C**) in the patient’s peripheral blood (PB) returned to almost normal after nine days of the first CD19 CAR T-cell infusion. The CRP (**Figure 5B**) decreased gradually from 114.26 mg/L to 0.80 mg/L after two weeks of the first CD19 CAR T-cell infusion.

Twelve weeks after the first CD19 CAR T-cell therapy, CD30 CAR T-cell were infused intravenously after partial myeloablation chemotherapy. Because the patient’s tumor loading was significantly reduced after CD19 CAR T-cell therapy, the incidence of severe adverse reactions was significantly reduced. The patient was given CD30 CAR T-cell therapy at a dose of 1.32×10^6^ cell/kg. After CD30 CAR T-cell infusion, lab results showed that IL-6 (**Figure 5E**) changed from 4.13 to 11.39 pg/ml, and hemoglobin and platelets (**Figure 5F**) were normal. There were no CD30 CAR T-cell detected in PB fifty days after CD30 CAR T-cell infusion.

After CD30 CAR T-cell infusion, the splenomegaly continued shrinking(**Figure 3D**) to CR, and CT scans showed spleen and lung metastases (**Figure 4A** 2018.10.22), and lymphadenopathy in the neck, abdomen (**Figure 4B** 2018.10.22), and pelvis continued shrink to CR in four monthes.

Serious acute toxicity after CAR T-cell infusion was not observed at any CD19 or CD30 CAR T-cell doses. The patient developed leukopenia as expected with cytotoxic chemotherapy. Labwork showed that the patient’s aspartate transaminase (AST) (**Figure 5D**) level was 7.94 times normal but then gradually decreased after three days of the third CD19 CAR T-cell infusion.

## Discussion

Intraclonal genetic diversity is a common feature of cancer and is the basis for clonal evolution, disease progression, relapse, and metastasis (19). An important consideration for therapeutic targeting is subclonal genetic complexity (19). As shown in the IHC staining in paraffin-embedded specimens, the RS cells of cHL expressed different antigens such as CD19, CD20, and CD30. These receptors might be potential targets for CAR T-cell therapy. Although the CAR T-cell therapy is effective, clonal cancer cells with other positive targets may relapse due to limitations of the CAR.

To understand which CAR T-cell could be used for the treatment of cHL, and how many cHL patients could receive multiple CAR T-cell therapy, we detected the expression of CD19, CD20, and CD30 in RS cells by IHC staining in 38 paraffin-embedded specimens from patients with cHL. Although most patients expressed CD20 and CD30, only 2 (5.2%) patients had positive expression of CD19 in RS cells. This suggests the limited use of CD19 CAR T-cell for treatment.

CD19 plays an indispensable role in regulating normal B-cell physiological response functions (20). B-cell related diseases are often linked to abnormal expression of CD19, and diminished CD19 expression is closely associated with B-cell related lymphomas ranging from chronic lymphocytic leukaemia (CLL), follicular lymphoma, and diffuse large B-cell lymphoma (20). The question remains whether tumor patients with abnormal B cells expression can be cured or at least benefit in their treatment of the tumor if the abnormal B cells are removed and the normal B cells are rebuilt.

In two previous r/r cHL clinical trials, positively expressed CD30 RS cells were confirmed by IHC; however, the percentage of positive cells were not detected in metastatic lymphoma by FCM. In one clinical trial, only one of four patients achieved CR after two infusions of CD30 CAR T-cell and another patient infused once with CD30 CAR T-cell had no response at a dose of 2×10^7^–10^8^ cells/m^2^ (2). In aother clinical trial, 18 patients received an infusion of a mean total of 1.56×10^7^/Kg CD30 CAR T-cell, where, 7 achieved partial remission and 6 achieved SD (3). The above clinical trials indicated that a single infusion of one type of CAR T-cell for cHL treatment is ineffective. In our patient with metastatic lymphoma, positively expressed CD30 RS cells were confirmed by IHC and the percentage of CD19 and CD30-positive cells detected by FCM were higher than CD20. Therefore, the patient received both CD19 and CD30 CAR T-cell treatment. Because our patient’s baseline characteristics were prone to severe adverse events, we attempted to reduce the risk of acute toxicities. We first treated the patient with three CD19 CAR T-cell infusions, which resulted in a good response. After CD19 CAR T-cell therapy, the patient’s tumor load was significantly reduced and severe adverse reactions did not occur. Then we gave the patient one high dose infusion of CD30 CAR T-cell. Once again the patient responded well and did not experience severe adverse events related to the therapy. She is currently in PFS for one year and three months at the time of this manuscript preparation.

The patient had many risk factors that could leed to CRS and/or NT, including high disease burden, severe thrombocytopenia, hyperpyrexia (38.5–41.0°C), blood platelet levels less than 60, and IL-6 higher than 16 pg/mLin the first 36 h after the CD19 CAR T-cell infusion (11). To reduce the risks of immediate toxicity, we used a low dose (1.61–5.20×10^5^cells/kg) of the CD19 CAR T-cell with three infusions at six-day intervals. Although the IL-6 level in the PB was temporarily elevated (180.37 pg/ml) in the first 42 h post-CD19 CAR T-cell infusion, it gradually decreased. There is no severe adverse reactions occur. Low dose CAR T-cell infusion may reduce the cytotoxicity of CAR T-cell to target cells and avoid the rapid release of cytokines in PB.

We showed that the first CD19 CAR T-cell treatment reduced the enlarged spleen, which could be attributed to the elimination of RS cells by CD19 CAR T-cell. The rapidly reduced spleen mass may have increased hemoglobin and platelets in PB. Abnormality liver function may be caused by the toxic side effects and the rapid disintegration of cancer cells induced by the CD19 CAR T-cell.

CAR T-cell immunotherapy can be used for the treatment of patients with poor physical condition, high disease burden, and r/r cHL. Successive low doses of CAR T-cell might reduce toxic side effects. CD19 and CD30 CAR T-cell treatment might not be suitable for most cHL patients, however, more clinical trials need to investigate this therapy.

## Data Availability Statement

The raw data supporting the conclusions of this article will be made available by the authors, without undue reservation.

## Ethics Statement

The studies involving human participants were reviewed and approved by The Third Affiliated Hospital of Kunming Medical University. The patients/participants provided their written informed consent to participate in this study. The animal study was reviewed and approved by the Ethics Committee of The Third Affiliated Hospital of Kunming Medical University.

## Author Contributions

YX, XS, HY, and RCL conceived the study. XL, RLL, CG, and BZ provided the clinical materials. ZL, QF, YZ, and SL collected the data. XL, QM, and AC revised the manuscript. SD, JY, and LZ performed the immunohistochemical staining. JG and HD perfomed the Flow Cytometry. QT and ZHL interpreted the data. YX wrote the manuscript. All authors read and approved the final version of the manuscript. All authors contributed to the article and approved the submitted version.

## Funding

This study was supported in part of Science Innovation Foundation of Guangdong Province, China (No. cxzd112). Science and Technology Program of Yunnan Province (No. 2018NS0050), and National Natural Science Foundation of China (No. 81660455).

## Conflict of Interest

AHC is a founding member of Shanghai YaKe Biotechnology Ltd, a biotechnology company focused on research and development of tumor cellular immunotherapy. YZ and SL are also employees of Shanghai YaKe Biotechnology Ltd.

The remaining authors declare that the research was conducted in the absence of any commercial or financial relationships that could be construed as a potential conflict of interest.
